# Atrial Fibrillation: Focus on Myocardial Connexins and Gap Junctions

**DOI:** 10.3390/biology11040489

**Published:** 2022-03-23

**Authors:** Yu-Han Guo, Yi-Qing Yang

**Affiliations:** 1 Department of Cardiology, Shanghai Fifth People’s Hospital, Fudan University, Shanghai 200240, China; dangochan@126.com; 2 Cardiovascular Research Laboratory, Shanghai Fifth People’s Hospital, Fudan University, Shanghai 200240, China; 3 Center Laboratory, Shanghai Fifth People’s Hospital, Fudan University, Shanghai 200240, China

**Keywords:** gap junctions, connexins, atrial fibrillation

## Abstract

**Simple Summary:**

Atrial fibrillation (AF) represents a worldwide medical problem contributing to substantial morbidity, mortality and social-economic burden. Gap junctions and their constituent components, connexins, are involved in the pathogenesis of AF, though the specific mechanisms have not been fully elucidated. We reviewed the current knowledge on the roles of connexin distribution and abundance in the pathogenesis of AF and the AF-related connexin mutations and polymorphisms as well as their pathogenic mechanisms. We also summarized the potential therapeutic targets and introduced connexin gene therapy for AF.

**Abstract:**

Atrial fibrillation (AF) represents the most common type of clinical cardiac arrhythmia worldwide and contributes to substantial morbidity, mortality and socioeconomic burden. Aggregating evidence highlights the strong genetic basis of AF. In addition to chromosomal abnormalities, pathogenic mutations in over 50 genes have been causally linked to AF, of which the majority encode ion channels, cardiac structural proteins, transcription factors and gap junction channels. In the heart, gap junctions comprised of connexins (Cxs) form intercellular pathways responsible for electrical coupling and rapid coordinated action potential propagation between adjacent cardiomyocytes. Among the 21 isoforms of connexins already identified in the mammal genomes, 5 isoforms (Cx37, Cx40, Cx43, Cx45 and Cx46) are expressed in human heart. Abnormal electrical coupling between cardiomyocytes caused by structural remodeling of gap junction channels (alterations in connexin distribution and protein levels) has been associated with enhanced susceptibility to AF and recent studies have revealed multiple causative mutations or polymorphisms in 4 isoforms of connexins predisposing to AF. In this review, an overview of the genetics of AF is made, with a focus on the roles of mutant myocardial connexins and gap junctions in the pathogenesis of AF, to underscore the hypothesis that cardiac connexins are a major molecular target in the management of AF.

## 1. Introduction

Atrial fibrillation (AF) is the most common cardiac rhythm disturbance, currently afflicting approximately 46.3 million individuals worldwide [1]. The prevalence of AF exponentially increases with age and reaches up to roughly 9% in individuals older than 80 years of age [2]. Comorbidities of AF substantially contribute to morbidity, mortality and medical care expenditure. The disease leads to severe major adverse cardiovascular events [3] and roughly 15% of all strokes [4]. AF is also associated with a three-fold increased risk for heart failure and a two-fold increased risk for all-cause mortality that is independent of comorbidities [5]. Nevertheless, the mechanisms causing and sustaining AF are multifactorial and incompletely understood. At present, it is still a great challenge to terminate AF.

AF is characterized by rapid and erratic atrial electrical activity, resulted from dynamic interplays among multiple electrophysiological, structural, inflammatory and genetic factors [6]. The disease is frequently secondary to diverse cardiovascular and systemic diseases such as hypertension, myocardial infarction, valvular dysfunction, heart failure and diabetes [7]. However, 2~16% of overall AF are defined as idiopathic AF for not having any identifiable underlying conditions [8]. Furthermore, approximately 30% of AF cases are of familial patterns [9], implying the genetic basis of AF. All these findings have provided substantial new insights into the mechanisms of AF. Up to now, mutations in over 50 genes have been causally linked to AF, most of which encode ion channels, structural proteins, transcription factors and gap junction channels [10]. 

Cardiac gap junctions are located at the intercalated disks between cardiomyocytes, forming intercellular pathways for the orderly propagation of electrical activation responsible for synchronized myocardial contraction [11]. The normal cardiac rhythm fundamentally depends on the cell-to-cell electrical coupling of cardiomyocytes through gap junctions. Therefore, it is possible that dysfunction or impairment of gap junctions my lead to cardiac arrhythmias. Indeed, a large amount of research has associated gap junctions and their structural components, connexins, with AF susceptibility. In this review, we aimed to outline the genetics of AF, focusing on the roles of mutant myocardial connexins and gap junctions in the pathogenesis of AF, to support the hypothesis that cardiac connexins are a major molecular target in the management of AF.

## 2. The Genetics of AF

The general hypothesis for the pathogenesis of AF is the combination of a vulnerable atrial substrate and initiating triggers. The triggered activities originated mainly from the pulmonary veins which promote reentrant waves and ectopic activities on the basis of abnormal calcium handling [12]. On the other hand, the perpetuation of AF is dependent on the atrial arrhythmogenic substrate which is characterized by regional conduction heterogeneity in atrial tissue [13]. Reentry, facilitated by short effective refractory period (ERP) or slow conduction velocity, seems to be the most probably mechanism of AF perpetuation [12,14].

To date, numerous AF-causing genes and risk loci have been identified, including those encoding ion channels, transcription factors, cardiac structural proteins and gap junction channels [15]. Each phase of the cardiac action potential is initiated and sustained by specific ion channels that generate ion currents and guarantee the normal electrophysiological properties of cardiomyocytes. As a result, gain-of-function or/and loss-of-function mutations in ion channel genes were firstly causally linked to AF, including different potassium channels, sodium channels and calcium channels as well as their auxiliary subunits [12]. The cardiac transcription factors are involved in both cardiogenesis and adult cardiac remodeling. Recently, increasing studies focusing on transcription factors have revealed series of variations which may predispose to AF by altering their transcriptional activities or/and synergistic transcriptional activities with their transcriptionally cooperative partners [10,15,16,17,18,19,20]. Mutations in NPPA (natriuretic peptide precursor A) and other genes involved in atrial fibrosis have been reported to enhance AF susceptibility by their contribution to the atrial structural remodeling [21] while mutations in nuclear structural protein genes by the disruption of nucleocytoplasmic transportation [22]. In addition, myocardial connexins and gap junctions have long been the targets of AF-related research for their critical roles in the cell-to-cell electrical coupling and impulse propagation. The detailed mechanisms will be elucidated below.

## 3. Structure and Function of Gap Junctions

**Figure 1:**
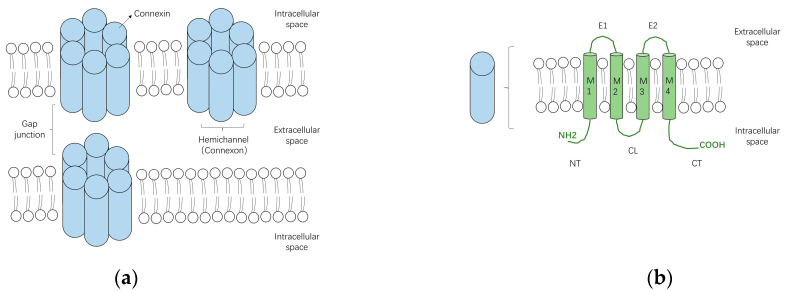
The schematic diagrams of gap junctions, hemichannels and connexins. (**a**) Two connexons from neighboring cells, which are formed by the oligomerization of six connexin subunits, can assemble into a gap junction. Connexons may also function as hemichannels under specific conditions; (**b**) Connexins are transmembrane proteins constituted by four transmembrane domains (M1–4) and intracellular N- and C-terminus (NT and CT), linked by two extracellular loops (E1 and E2) and one cytoplasmic loop (CL).

Gap junctions are clusters of transmembrane channels that enable the direct cytoplasmic exchange of ions or small metabolites (<1 kDa in size) between neighboring cells. They are constructed by the juxtaposition of a pair of hemichannels (connexons) from adjoining cells. Under pathological conditions or specific circumstances such as paracrine signaling [23], hemichannels that span the entire depth of the plasma membrane may also function as transmembrane channels in unopposed cells, allowing the permeation of ions and small metabolites [24] (Figure 1a). Each hemichannel is formed by oligomerization of six connexins (Cxs) surrounding a central aqueous pore [25]. Connexins are tetra-span transmembrane domain proteins with four highly conserved transmembrane domains (M1–M4) and intracellular N- and C-terminus (NT and CT), linked by two extracellular loops (E1 and E2) and one cytoplasmic loop (CL) (Figure 1b). 

Practically all cells in solid tissues are linked by gap junctions and the majority of cells co-express more than one type of connexin [26]. Currently, 21 isoforms of connexin have been identified in the human genome and 20 in the murine genome. They are divided into five subfamilies (α, β, γ, δ and ε or GJA, GJB, GJC, GJD and GJE) according to sequence homology [27]. Identical Cxs in both docked hemichannels comprise homotypic gap junction channels while mixed Cxs constitute heteromeric gap junction channels. In addition, two different homotypic hemichannels make up a heterotypic gap junction channel. Particular connexin types or combination of connexin types in hemichannels leads to distinction in the physiological properties of gap junction channels [28]. 

The main functions of gap junctions are to share small nutrients or signaling molecules among groups of cells and permeate ions across electrically excitable cells to coordinate electrical and mechanical actions in tissues such as heart, neurons and smooth muscle [23]. Gating of gap junction intercellular channels is dynamically regulated by multiple factors including transjunctional voltage, intracellular calcium concentration, pH, phosphorylation and other post-translational modifications [26].

## 4. Subtypes of Cardiac Connexins

To date, six principal connexins are found expressed in human heart (Cx37, Cx40, Cx43, Cx45 and Cx46) [14,29]. Cx43 is the predominant cardiac connexin which is mainly distributed in the atrial and ventricular cardiomyocytes [30]. It is less expressed in the conduction system and not expressed in the sinoatrial or atrioventricular node [30,31]. Cx40, another significant cardiac connexin, is restricted primarily to the atrial tissue and the ventricular conduction system and is 2~3 fold higher in the right atrium than the left [14,31]. Cx45 is the first connexin expressed during early stages of cardiovascular development [30]. However, in the adult heart, it is expressed predominantly in the conduction system while expressed in low quantities in both ventricles and atria with a slightly higher level in the atrium than the ventricle [32]. Cx37 is expressed mainly in the vascular endothelium [31]. In addition, the less studied cardiac connexin Cx46 has been found in the atrial and ventricular myocytes in the human heart [33].

The biologic functions of these cardiac connexins are currently being elucidated. In addition to allowing rapid propagation of action potentials mediating coordinated myocardial contraction in the adult heart, these connexins are crucial for heart development through mediating the exchange of critical factors between cells. In neonatal mice, Cx43 knockout leads to death at birth because of right ventricular outflow tract malformations [34]. It was also reported that cardiac malformations are prevalent in Cx40-deficient mice [35]. Likewise, lacking Cx45 in mice predisposes to embryonic death for sinus node dysfunction and atrial arrhythmia [36]. In addition, several recent studies have linked Cx37 deficiency to both venous and lymphatic valve malformation [37,38].

## 5. Changes of Gap Junctions/Connexins in the Pathogenesis of AF

### 5.1. Gap Junction Remodeling

The process of gap junction remodeling, characterized by alterations in gap junction channel abundance, subcellular distribution, permeability (determined by the phosphorylation status of the constituent connexins) and conductance [39], is an important portion of the heart adaptive remodeling during cardiac diseased states. It is associated closely with cardiac electrical remodeling which leads to alterations in conduction [40]. The impaired gap junction intercellular communication is increasingly observed in human cardiovascular diseases such as heart failure, ischemic heart disease and cardiac arrythmias [28]. Nevertheless, the explicit relationship between gap junction remodeling and the pathogenesis of these diseases still needs further clarifications. 

As mentioned above, cardiac gap junction channels play crucial roles in direct intercellular communication and myocardial synchronization in adult heart. Accordingly, any form of cardiac gap junction remodeling may disturb these functions and thus contribute to arrhythmia vulnerability. In fact, the onset of arrhythmias involves the interaction of gap junction intercellular communication, cell membrane excitability and the structures of cell and tissue [28]. Ventricular myocytes contain predominantly Cx43 isoform except for rather low levels of Cx45 and Cx46 while multiple isoforms of connexins (Cx40, Cx43 and Cx45) are expressed in the atria, the most connexin-heterogeneous tissue of the heart [14], causing diversity in types and physiological characteristics of atrial gap junctions. That is to say, it is much more difficult to figure out the patterns of gap junction remodeling of atrial arrhythmias than ventricular arrythmias.

### 5.2. Abundance and Distribution of Connexins Associated with AF

Ever since Spach et al. [41] first highlighted alterations in gap junction structure and function as potential therapeutic targets for AF, a large number of studies have been conducted on the role of connexins in the pathogenesis of AF [42]. Most of these studies focused on changes in the abundance and distribution of connexins in the AF models or patients.

Changes in Cx40/Cx43 quantity caused by AF are uncertain because of the inconsistency of the results. The abundance of Cx43 seems to be dependent on types of AF while the amount of Cx40 caused by AF may increase with Cx40 lateralization, or reduce significantly, or be indistinguishable from sinus rhythm, or be dependent on extracellular Ca^2+^ level [25]. Wetzel and his colleagues [43] found increases in both Cx40 and Cx43 concentration in left atrial tissue of lone AF patients and AF patients with mitral valve disease, when compared with sinus rhythm. In another study aiming at chronic AF [44], the authors observed no significant change in Cx43 content in patients’ atrial tissues while Cx40 was enhanced. Moreover, Kanagaratnam et al. [45] observed a reduction of Cx40 in chronic AF with complex activation.

**Figure 2:**
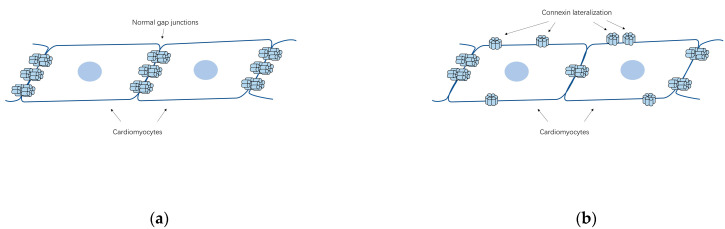
The schematic diagrams of the lateralization of cardiac connexins in AF. (**a**) Schema of the cell-to-cell connections by gap junctions in normal heart; (**b**) The lateralization of connexins from cell poles to lateral margins, which can be related to AF.

Apart from changes in their quantity, the lateralization of Cx40/Cx43 from cell poles to lateral margins (Figure 2b) appears to be a general AF-associated alteration. In Polontchouk and his colleagues’ research [44], an increase in both Cx40 and Cx43 at the lateral membrane of human and rat atrial cells was found, indicating that AF might be accompanied by the spatial remodeling of gap junctions. Similar results were observed by Kostin et al. afterwards [46]. In their research, lateralization of Cx43, Cx40 and N-cadherin, reduction of Cx43 level and heterogeneous distribution of Cx40 together with augmentation of fibrosis were found in AF patients, constituting the anatomic substrates of AF [46]. In addition, Dhein et al. detected AF induced Cx40/Cx43 lateralization together with enhanced lateral conduction velocity in the left atrial [47]. These redistributed connexins lost their characteristic to assemble into gap junctions coupling with adjacent cells but may function as hemichannels which allow ionic currents and small metabolites [48,49]. It remains unclear what are the main factors that lead to the spatial remodeling of the gap junctions. However, AF causes a rather complicated intracellular remodeling which may constitute the basis for the redistribution of gap junctions and the intracellular remodeling of the Golgi-microtubular apparatus, where the connexins oligomerize, is also included [50]. In fact, the fragmentation and a decreased fragment size of the Golgi apparatus were observed in patients with chronic AF [51].

In brief, the pathogenesis of AF may be associated with the lateralization of cardiac connexins but the role of connexin abundance remains indistinct.

### 5.3. Changes in Atrial Connexin Expression Regulated by Transcription Factors

Abnormal transcriptional regulation of gene expression may be a characteristic feature in the occurrence of heart diseases. Transcription factors regulating cardiac connexins can be roughly classified into two categories based on the ubiquity or cardiac cell specificity of their binding sites. The former include Sp1/Sp3 and activator protein 1 (AP-1) while the latter include cardiac specific transcription factors such as NKX2-5, Shox2, T-box transcription factors and GATA family [52]. Some of these transcription factors might contribute to AF through regulating the expression of atrial connexins.

Ma et al. [53] sequenced a series of candidate genes in 139 Chinese patients with early-onset AF and found four missense TBX5 mutations. Subsequent functional experiments indicated that these mutations increased the expression of Cx40 and NPPA without altering the expression of cardiac structural protein genes in rat atrial myocytes. In the zebrafish model, the overexpression of TBX5 p.R355C mutation caused paroxysmal AF. Later in 2019, a gain-of-function mutation of the paired-like homeodomain transcription factor 2 (PITX2) were identified by Mechakra et al. in 1 out of 60 unrelated idiopathic AF patients [54]. The mutation was then introduced into HL-1 cells and increased mRNA level of GJA5 (coding for connexin 40, 3.1-fold increase) and GJA1 (coding for connexin 43, 2.1-fold) was observed. 

In addition, phosphorylation of transcription factors can also affect connexin expression. For example, the activation of c-Jun (AP-1) N-terminal kinase has been reported to cause decreased Cx43 level in HL-1 cells [55]. Reduced conduction velocity and increased incidence of irregular rapid spontaneous activities that may contribute to AF were also observed.

## 6. Mutations of Connexins Associated with AF

The prevailing postulate “AF begets AF” summarized the self-stabilizing characteristic of AF [56]. In other words, AF begins with paroxysmal attacks, which gradually increases in frequency and duration and finally progresses into more persistent AF subtypes. The remodeling of intercellular coupling plays an important part in this process. 

More and more researchers have recognized the mutations of cardiac connexins as a significant AF substrate although their incidence appears to be rather low. In this case, the regional decrease in the gap junction coupling conductance between myocardial cells leads to increased heterogeneity in cardiac conduction velocity and eventually contributes to reentry, which is the most important mechanism of AF [57,58]. Up to now, multiple mutations in cardiac Cx40 (GJA5) have been causally linked to AF through different mechanisms. For Cx43 (GJA1) and Cx45 (GJC1), however, they each have only a single report of a mutation potentially related to AF. Further extensive studies are still needed to confirm their association with AF. In addition, a polymorphism of GJA4 (Cx37) was recently associated with AF. Cx46 (GJ13) may play a crucial role in the physiopathology of heart failure but has nothing to do with AF according to current research [29]. 

### 6.1. Cx40 (GJA5) Mutations and AF

Cx40 is encoded by the GJA5 gene, which is located on chromosome 1q21.2. Unlike Cx43, which is heterogeneously distributed, Cx40 is mainly expressed in the atria and is the predominant connexin for atrial impulse conduction [25]. It was reported that Cx40-deficient mice presented significant electrocardiographic changes including a prolonged P wave, PQ or PR interval, QTc and QRS duration and were more susceptible to atrial tachyarrhythmias [59]. Nevertheless, the propagation velocity in human atria is associated with the interactions between Cx40 and Cx43 expression which may result in novel coupling properties [60]. This can be explained by the fact that in the atria, heterotypic Cx40/Cx43 gap junction channels have much lower conductance than Cx40 or Cx43 homotypic gap junction channels [61]. For AF patients, the distribution of Cx40 is predominantly lateralized within myocardial cells [44,46] and heterogeneous in the atria [46,62], which may lead to a heterogeneous cell-to-cell coupling and disturb the normal pattern of coordinated myocardial excitation thereby promoting reentry [63]. In human studies, variants in the promoter or regulatory regions of GJA5 could reduce the expression level of Cx40 and have been linked to AF [64,65,66,67,68]. Furthermore, three somatic mutations and ten germline mutations in the coding region of the GJA5 gene have been identified in recent years. 

In 2006, Gollob and his colleagues identified four novel GJA5 heterozygous missense mutations from 15 patients with idiopathic AF, which were G38D, P88S, A96S and M163V [69]. Three of the mutations were somatic mutations which were found only in the cardiac specimens while A96S was found in both cardiac tissue and blood leucocytes, indicating a germ-line origin. Functional analyses on the mutant Cx40 showed impaired gap junction assembly or/and reduced cell-to-cell electrical coupling, which may predispose the atria to AF. The AF-related A96S mutation was also identified in another investigation in which the authors sequenced the coding region and flanking introns of GJA5 in 342 patients with early-onset lone AF [67]. At the same time, a significant association between the A allele of rs10465885 in GJA5 and AF was also found. Interestingly, a study introduced the A96S mutation of Cx40 into mice and generated a model for AF. The mice showed reduced atrial conduction velocity and sustained durations of induced AF [70].

Next, a germ-line mutation in GJA5 gene, Q49X, was identified in a family with idiopathic AF by Yang et al. [71]. The novel nonsense mutation, predicted to introduce a premature stop codon at amino acid 49, caused the truncation of Cx40. It co-segregated with AF in the family in an autosomal-dominant way but was not found in the unaffected relatives and the control individuals. In a follow-up research, Sun et al. performed functional analyses on the Q49X mutant and proved that the mutation disturbed the intracellular distribution and the cell-to-cell electrical coupling of gap junctions and also impaired the localization of both wild-type Cx40 and Cx43 to the cell-to-cell interfaces, which might be the pathogenesis of AF in the family [72]. 

Further, Yang et al. sequenced the entire coding region of GJA5 from the genomic DNA of 218 unrelated probands with familial AF and identified 3 novel germline heterozygous mutations (V85I, L221I and L229M) that co-segregated with AF in the probands’ respective family [73]. All the mutations were predicted to be disease-causing and the altered amino acids caused by these mutations were completely conserved evolutionarily. The pathogenic mechanisms of these mutations were studied by Sun and his colleagues subsequently [74,75]. It was reported that the AF-linked L229M mutation significantly reduced the gap junction function when expressed together with wild-type Cx43 but did not impair coupling conductance when expressed alone or co-expressed with wild-type Cx40 [74]. The other two Cx40 mutants, V85I and L221I, however, were shown to increase hemichannel function but not impair the gap junction coupling [75]. In their study, Sun et al. discovered both V85I and L221I expressing cells showed significant propidium iodide (PI)-uptake while wild-type Cx40 and other AF-linked mutants failed to present it. What’s more, the PI-uptake was sensitive to [Ca^2+^] and the hemichannel blockers but not affected by the pannexin 1 channel blocking agent, indicating the PI uptake was most likely mediated by connexin hemichannels. The gain-of-function hemichannels in the two AF-related Cx40 mutants may provide a brand-new possible mechanism for the pathogenesis of AF. 

In 2013, Sun et al. sequenced the whole coding region and splice sites of GJA5 in 68 unrelated patients with lone AF and identified a novel germline missense mutation I75F in 1 patient [74]. This Cx40 mutation was also present in the proband’s father with lone AF, but absent in the unaffected family members and the control individuals. In addition, electrophysiological studies indicated that I75F did not constitute functional gap junction channels when expressed alone and damaged the gap junction coupling conductance when co-expressed with wild-type Cx40 or Cx43, which might play a role in the onset of AF in the mutation carriers. 

Similarly, 4 novel heterozygous GJA5 mutations, K107R, L223M, Q236H and I257L, were identified in 4 of 310 unrelated patients with lone AF [76]. The mutations were present in all the affected relatives of each carrier’s family, respectively, but absent in the controls. In addition, the amino acids altered by these mutations except I257L were highly conserved among species. These findings expand the spectrum of Cx40 mutations related to AF and provide new insights into the molecular etiology of AF. The functional characterization of these AF-linked GJA5 mutations were performed by Noureldin et al. in 2018. As was shown in the results, K107R, L223M and I257L did not affect gap junction localization, function or hemichannel activities and therefore the relationship between AF and these mutants remains unclear [77]. In contrast, Q236H exhibited a significantly reduced gap junction coupling conductance when expressed alone or co-expressed with wild-type Cx43. In addition, Q236H gap junctions also presented altered transjunctional voltage-dependent gating. These defects related to Q236H might predispose the mutation carriers to AF.

Generally speaking, these AF-linked Cx40 mutants present either an impairment of gap junction function or a gain of Cx40 hemichannel function, but the underlying molecular mechanisms seem to be quite different (Table 1). The mutants that impair gap junction channel function may adversely affect the cell-to-cell electrical coupling and result in heterogeneous conduction in the atrial tissue, providing a substrate for AF. The opening of hemichannels might alter the electrical properties of myocardial cells in several ways and thereby promote AF substrate. Firstly, the enhanced hemichannel function might allow Na^+^ influx and K^+^ efflux according to their electrochemical gradient and results in membrane depolarization, which may lead to the inactivation of Na^+^ channels. As a result, the heterogeneity of cardiomyocytes in excitability and conduction velocity may predispose the atria to AF [78]. Secondly, the open hemichannels might allow for leakage of small signaling and metabolic molecules which could be essential for normal cardiomyocyte function. Abnormalities of the atrial myocytes together with other vulnerable atrial substrates contribute to the perpetuation of AF by stabilizing reentry [12]. In addition, the intracellular ATP might also be released through the open hemichannels, causing intracellular Ca^2+^ wave propagation through purinergic receptors. This abnormal electrical activity of cardiomyocytes may also contribute to AF substrate [79].

### 6.2. Cx43 (GJA1) Alterations Implicated in AF

The GJA1 gene resides on chromosome 6q22-q23 and encode Cx43, which is the most widely expressed connexin and has been found in at least 35 cell types and tissues [59]. Mutations in Cx43 were found to be related to oculodentodigital dysplasia (ODDD) in the first place [80]. Patients with ODDD present an autosomal dominant syndrome of developmental malformations in their eyes, teeth, noses, digits and other defects including heart defects in some instances [81,82]. However, most of the ODDD patients with a variety of mutations in Cx43 do not exhibit cardiac arrhythmias [83].

Afterwards, a non-ODDD-associated Cx43 mutation was reported in AF. Thibodeau et al. [84] identified a loss-of-function mutation of GJA1 (c.932delC) from the atrial tissue of a sporadic AF patient. Electrophysiological studies proved no electrical coupling in the cells expressing the mutant Cx43 alone and significantly reduced coupling when co-expressing with wild-type Cx43 and Cx40 [84]. The findings altogether indicate that the Cx43 frameshift mutation contributes to heterogeneous coupling patterns in the atrium that predispose to AF.

### 6.3. Cx45 (GJC1) Mutations and Complex Arrhythmias

The GJC1 gene encoding Cx45 is located on chromosome 17q21.3. As mentioned above, Cx45 is mainly involved in the early stage of cardiovascular development and is characterized by low abundance in the adult heart [85]. Unlike Cx40 and Cx43, the fact that Cx45 knock-out animals die after birth [86] makes Cx45 less explored. However, Cx45 is proved not to be essential for the viability of adult mice but is required for optimal atrioventricular nodal conduction in the adult mouse heart [87]. Despite its low level of expression, Cx45 oligomerizes with the ventricular Cx43 and the atrial Cx40 and Cx43, forming functional heteromeric or heterotypic gap junctions. More importantly, the heteromeric gap junctions assembled by Cx40 and Cx43 appear to form only when Cx45 is present [88]. In a human study, a loss-of-function mutation in GJC1, R75H, was identified in two unrelated families with progressive atrial conduction system defects, including atrioventricular block (AVB) and atrial standstill [36]. Furthermore, the cardiac-specific Cx45 knockout mice presented similar intra-atrial disorders, indicating the potentially crucial role of Cx45 in the atrium. 

Recently, Li et al. conducted a genome-wide scan as well as a whole-exome sequencing analysis on a large Chinese family with autosomal-dominant AF and other cardiac arrhythmias encompassing AVB, sinus bradycardia and premature ventricular contractions [89]. As a result, a novel heterozygous loss-of-function mutation, M235L, in the GJC1 gene was identified in all the affected family members and was not found in 632 controls. The functional characterization of M235L indicated impaired subcellular localization and decreased transjunctional coupling conductance in cell pairs with Cx40 or Cx43. Therefore, the dysfunction of gap junction caused by the M235L mutant may provide a substrate for multiple wavelet reentry, which has generally been regarded as the main pathogenesis of AF.

### 6.4. A GJA4 (Cx37) Polymorphism and Non-Structural AF

The GJA4 gene encodes Cx37 and resides on the human chromosome 1p34.3. Apart from the vascular endothelium, Cx37 is also found in oocytes, monocytes/macrophages and platelets [59]. The single nucleotide polymorphism (SNP) C1019T of GJA4 leads to a substitution of proline to serine at amino acid 319 in Cx37. It was first described by Richard et al. [90] and was verified to change gap junction channel conductance and permeability [91]. Nevertheless, Kumari et al. [92] suggested that it was not the polymorphic variants but the amino terminus of Cx37 that played an important role in gating and conductance. 

The Cx37 C1019T polymorphism has been linked to a variety of human diseases including coronary heart disease (CHD), peripheral artery disease, in-stent restenosis, myocardial infarction (MI) and stroke [93,94,95,96,97], most likely through regulating the adhesion of monocytes [98]. In a meta-analysis, Wen et al. [99] enrolled a total of 3498 MI patients and 3986 controls from PubMed, Embase and Cochrane library. The overall odds ratios (OR) and 95% confidence intervals (95% CI) were 1.04, 0.95–1.15; and 1.02, 0.85–1.22 in dominant and recessive models, respectively. Another meta-analysis aiming at evaluating the role of C1019T in the pathogenesis of CHD was conducted by Zhao and his colleagues [100]. They included 9 case-control studies with a total of 1426 CHD cases and 929 controls and calculated the ORs and their 95% CIs. For T allele vs. C allele, OR = 1.63, 95% CI = 1.20–2.21, *p* = 0.002, indicating that C1019T may contribute to the pathogenesis of CHD.

In 2018, Carballo et al. reported for the first time that the Cx37 C1019T polymorphism was associated with drug-resistant non-structural AF rather than structural AF with underlying cardiomyopathy [68]. The authors speculated that the C1019T variant might enhance the susceptibility to unstructured AF through two mechanisms: firstly, the variant affected the adhesion of monocytes to modulate the extent of local inflammation, which was increasingly recognized to be involved in the onset of AF; secondly, Cx37 was also expressed in vascular smooth muscle cells at the orifice of pulmonary veins and the variant altered the electrophysiological properties of gap junction channels, which might disturb cardiac conduction and contributed to AF. All in all, further functional characterization of the Cx37 C1019T polymorphism is still needed.

## 7. Clinical Implications

As mentioned above, Spach et al. proposed in 1995 that altering the topology of gap junctions might be a therapeutic target for AF [41]. After years of research, the concept of therapeutics for AF based on the normalization of connexin expression and gap junction distribution has been widely recognized. It was reported that the AF-induced gap junctional remodeling could be reversed and this reversion was accompanied by a reduction in atrial susceptibility to AF [31]. Furthermore, multiple studies have demonstrated that the connexin function can be modified [25]. However, little is known about the factors that specifically regulate cardiac connexin function during AF.

Nowadays, a novel antiarrhythmic agent, an antiarrhythmic peptide (AAP), has been described to improve gap junctional conductance with antiarrhythmic potential [101]. The enhancement of intercellular coupling seems to be based on a PKC controlled phosphorylation of gap junctions [31]. ZP123, also known as rotigaptide, is an AAP that has been developed. It was reported that ZP123 improved conduction in multiple models of AF but its antiarrhythmic efficacy seemed to be limited to the ischemia-induced AF model [102].

As reported in a recent study, the Cx43 hemichannel blockade was screened as a potent inhibitor for the abnormal phenotypes in both hESC-atrial cells in vitro model and zebrafish in vivo model of MYL4 (myosin light chain 4) mutation related AF [103]. MYL4 was causally linked to AF in a previous study by Orr et al. [104]. Further experiments by Ghazizadeh et al. revealed that the MYL4 mutations led to mislocation and enhanced permeability of CX43 hemichannel which finally predisposed to AF substrate in both models [103]. They ultimately proposed the connexin 43 hemichannel blockers (carbenoxolone, Gap19 and Gap26) and PKC inhibitors (blocking the phosphorylation of Cx43 hemichannels) as potential therapeutic targets for AF [103]. In 2011, a peptide mimetic of the Cx43 C-terminus functioning as a hemichannel blocker was found to decrease gap junction remodeling and thus reduced inducible-arrhythmia following injury in mice [105].

In addition, the mimetic peptides, other modifiers of Cx43 have been found. Salameh et al. [106] reported that the cyclic mechanical stretch induced changes in expression and polarization of Cx43, which could be regulated by angiotensin II. The AT(1)-receptors enhance Cx43 expression while the AT(2)-receptors decrease Cx43 lateralization. It is known that both cyclic mechanical stretch and angiotensin II play crucial roles in cardiac remodeling including AF [107]. Nevertheless, the interplay between angiotensin II and Cx43 in AF patients, which may provide new insights into the therapeutic targets of AF, has not been studied yet. Beta-adrenoceptor stimulation, the activation of which is a hallmark of heart failure, has been proved to reduce the Cx43 expression and enhance the lateral distribution in cardiomyocytes, which is related to the inverse effects in cardiac fibroblasts [108]. Correspondingly, the pharmacological effect of metoprolol, a selective beta-blocker, on gap junction remodeling in human chronic AF has been identified [47]. Specifically speaking, the AF induced enhanced lateral expression of Cx43 together with increased transverse conduction velocity in the left atrial tissue could both be antagonized by metoprolol [47]. In addition, a study reported that in murine ventricular myocardial cells, the activation of ryanodine receptors (RyRs) by caffeine could induce an inward current which was attributed to Cx43 hemichannel openings [109]. The same results were observed in atrial myocytes from human and mice [49]. That is to say, RyRs agonist has the potential to act as a Cx43 hemichannel blockade and plays its role in AF management but further substantial research is needed.

Apart from the modifiers of cardiac connexins, the connexin gene therapy is another potential therapeutic for AF. Igarashi et al. reported a method for normalizing connexin expression using adenovirus expressing Cx40 or Cx43 in a porcine model of AF [110]. In their study, the expression of Cx43 was reduced and lateralized in AF animal models and the gene transfer of Cx43 normalized Cx43 expression and localization to sinus rhythm levels. In addition, both Cx40 and Cx43 gene transfer improved atrial conduction and reduced AF susceptibility compared with the controls. The study is a significant advance in both pathophysiology and therapeutics of AF, indicating the critical roles that connexins play. However, there are huge obstacles to applying cardiac connexin gene therapy to humans.

## 8. Conclusions

The genetics of AF is rather complicated and it becomes much more complicated when myocardial connexins and gap junctions are involved. Mutations in different cardiac connexins and even different mutations in the same connexin affect the function of gap junctions or hemichannels through various mechanisms leading to increased susceptibility to AF. 

Nowadays, more and more gene mutations and genetic polymorphisms of myocardial connexins have been linked to AF. However, most of these studies did not provide enough molecular mechanisms underlying the pathogenesis of AF. Therefore, studying on links between pathologies and mutations is as important as investigating the molecular and cellular mechanisms. In addition, more detailed and systematic studies on each AF-linked mutation are needed to develop effective strategies to restore normal gap junction/hemichannel function. Research on druggable molecular pathways related to myocardial connexins is also required as connexins might be a type of promising therapeutical targets for AF based on existing research.

## Figures and Tables

**Table 1:** Summary of functional characteristics of AF-linked myocardial connexin mutants.

AF-Linked Mutations		Gap Junction Function			Hemichannel Function
		Mutant Alone	Mutant on Wild-Type Cx40	Mutant on Wild-Type Cx43	
Cx43	c.932delC	eliminated	reduced	reduced	not tested
Cx40	G38D	reduced	not tested	not tested	not tested
	P88S	eliminated	reduced	reduced	not tested
	A96S	reduced	reduced	reduced	not tested
	M163V	normal	not tested	not tested	not tested
	Q49X	eliminated	reduced	reduced	normal
	V85I	normal	not tested	normal	enhanced
	L221I	normal	not tested	normal	enhanced
	L229M	normal	normal	reduced	normal
	I75F	eliminated	reduced	reduced	normal
	K107R	normal	normal	normal	normal
	L223M	normal	normal	normal	normal
	Q236H	reduced	not tested	reduced	not tested
	I257L	normal	normal	normal	normal
Cx45	M235L	reduced	reduced	reduced	not tested

## Data Availability

The study did not report any novel data.

## References

[1] Benjamin E.J., Muntner P., Alonso A., Bittencourt M.S., Callaway C.W., Carson A.P., Chamberlain A.M., Chang A.R., Cheng S., Das S.R. (2019). Heart Disease and Stroke Statistics-2019 Update: A Report From the American Heart Association. Circulation.

[2] Go A.S., Hylek E.M., Phillips K.A., Chang Y., Henault L.E., Selby J.V., Singer D.E. (2001). Prevalence of diagnosed atrial fibrillation in adults: National implications for rhythm management and stroke prevention: The AnTicoagulation and Risk Factors in Atrial Fibrillation (ATRIA) Study. JAMA.

[3] Wijesurendra R.S., Casadei B. (2019). Mechanisms of atrial fibrillation. Heart.

[4] Fuster V., Ryden L.E., Cannom D.S., Crijns H.J., Curtis A.B., Ellenbogen K.A., Halperin J.L., Kay G.N., Le Huezey J.Y., Lowe J.E. (2011). American College of Cardiology Foundation/American Heart Association Task Force: 2011 ACCF/AHA/HRS focused updates incorporated into the ACC/AHA/ESC 2006 guidelines for the management of patients with atrial fibrillation: A report of the American College of Cardiology Foundation/American Heart Association Task Force on practice guidelines. Circulation.

[5] Zimetbaum P. (2017). Atrial Fibrillation. Ann. Intern. Med..

[6] Saffitz J.E. (2006). Connexins, conduction, and atrial fibrillation. N. Engl. J. Med..

[7] January C.T., Wann L.S., Alpert J.S., Calkins H., Cigarroa J.E., Cleveland J.C., Conti J.B., Ellinor P.T., Ezekowitz M.D., Field M.E. (2014). 2014 AHA/ACC/HRS guideline for the management of patients with atrial fibrillation: A report of the American College of Cardiology/American Heart Association Task Force on practice guidelines and the Heart Rhythm Society. Circulation.

[8] Campuzano O., Perez-Serra A., Iglesias A., Brugada R. (2016). Genetic basis of atrial fibrillation. Genes Dis..

[9] Fox C.S., Parise H., D’Agostino R.B., Lloyd-Jones D.M., Vasan R.S., Wang T.J., Levy D., Wolf P.A., Benjamin E.J. (2004). Parental atrial fibrillation as a risk factor for atrial fibrillation in offspring. J. Am. Med. Assoc..

[10] Guo X.J., Qiu X.B., Wang J., Guo Y.H., Yang C.X., Li L., Gao R.F., Ke Z.P., Di R.M., Sun Y.M. (2021). PRRX1 Loss-of-Function Mutations Underlying Familial Atrial Fibrillation. J. Am. Heart Assoc..

[11] Severs N.J., Dupont E., Thomas N., Kaba R., Rothery S., Jain R., Sharpey K., Fry C.H. (2006). Alterations in cardiac connexin expression in cardiomyopathies. Adv. Cardiol..

[12] Staerk L., Sherer J.A., Ko D., Benjamin E.J., Helm R.H. (2017). Atrial Fibrillation: Epidemiology, Pathophysiology, and Clinical Outcomes. Circ. Res..

[13] Gollob M.H. (2006). Cardiac connexins as candidate genes for idiopathic atrial fibrillation. Curr. Opin. Cardiol..

[14] Duffy H.S., Wit A.L. (2008). Is there a role for remodeled connexins in AF? No simple answers. J. Mol. Cell. Cardiol..

[15] Lozano-Velasco E., Franco D., Aranega A., Daimi H. (2020). Genetics and Epigenetics of Atrial Fibrillation. Int. J. Mol. Sci..

[16] Wang J., Zhang D.F., Sun Y.M., Yang Y.Q. (2014). A novel PITX2c loss-of-function mutation associated with familial atrial fibrillation. Eur. J. Med. Genet..

[17] Guo D.F., Li R.G., Yuan F., Shi H.Y., Hou X.M., Qu X.K., Xu Y.J., Zhang M., Liu X., Jiang J.Q. (2016). TBX5 loss-of-function mutation contributes to atrial fibrillation and atypical Holt-Oram syndrome. Mol. Med. Rep..

[18] Li N., Wang Z.S., Wang X.H., Xu Y.J., Qiao Q., Li X.M., Di R.M., Guo X.J., Li R.G., Zhang M. (2018). A SHOX2 loss-of-function mutation underlying familial atrial fibrillation. Int. J. Med. Sci..

[19] Wu S.H., Wang X.H., Xu Y.J., Gu J.N., Yang C.X., Qiao Q., Guo X.J., Guo Y.H., Qiu X.B., Jiang W.F. (2020). ISL1 loss-of-function variation causes familial atrial fibrillation. Eur. J. Med. Genet..

[20] Li N., Xu Y.J., Shi H.Y., Yang C.X., Guo Y.H., Li R.G., Qiu X.B., Yang Y.Q., Zhang M. (2021). KLF15 Loss-of-Function Mutation Underlying Atrial Fibrillation as well as Ventricular Arrhythmias and Cardiomyopathy. Genes.

[21] Cheng C., Liu H., Tan C., Tong D., Zhao Y., Liu X., Si W., Wang L., Liang L., Li J. (2019). Mutation in NPPA causes atrial fibrillation by activating inflammation and cardiac fibrosis in a knock-in rat model. FASEB J..

[22] Han M., Zhao M., Cheng C., Huang Y., Han S., Li W., Tu X., Luo X., Yu X., Liu Y. (2019). Lamin A mutation impairs interaction with nucleoporin NUP155 and disrupts nucleocytoplasmic transport in atrial fibrillation. Hum. Mutat..

[23] Goodenough D.A., Paul D.L. (2009). Gap junctions. Cold Spring Harb. Perspect. Biol..

[24] Saez J.C., Leybaert L. (2014). Hunting for connexin hemichannels. FEBS Lett..

[25] Chaldoupi S.M., Loh P., Hauer R.N., de Bakker J.M., van Rijen H.V. (2009). The role of connexin40 in atrial fibrillation. Cardiovasc. Res..

[26] Nielsen M.S., Axelsen L.N., Sorgen P.L., Verma V., Delmar M., Holstein-Rathlou N.H. (2012). Gap junctions. Compr. Physiol..

[27] Beyer E.C., Berthoud V.M. (2018). Gap junction gene and protein families: Connexins, innexins, and pannexins. Biochim. Biophys. Acta Biomembr..

[28] Severs N.J., Bruce A.F., Dupont E., Rothery S. (2008). Remodelling of gap junctions and connexin expression in diseased myocardium. Cardiovasc. Res..

[29] Ortega A., Tarazon E., Gil-Cayuela C., Garcia-Manzanares M., Martinez-Dolz L., Lago F., Gonzalez-Juanatey J.R., Cinca J., Jorge E., Portoles M. (2017). Intercalated disc in failing hearts from patients with dilated cardiomyopathy: Its role in the depressed left ventricular function. PLoS ONE.

[30] Desplantez T. (2017). Cardiac Cx43, Cx40 and Cx45 co-assembling: Involvement of connexins epitopes in formation of hemichannels and Gap junction channels. BMC Cell. Biol..

[31] van der Velden H.M., Jongsma H.J. (2002). Cardiac gap junctions and connexins: Their role in atrial fibrillation and potential as therapeutic targets. Cardiovasc. Res..

[32] Severs N.J., Coppen S.R., Dupont E., Yeh H.I., Ko Y.S., Matsushita T. (2004). Gap junction alterations in human cardiac disease. Cardiovasc. Res..

[33] Cheng S.H., Shakespeare T., Mui R., White T.W., Valdimarsson G. (2004). Connexin 48.5 is required for normal cardiovascular function and lens development in zebrafish embryos. J. Biol. Chem..

[34] Reaume A.G., Desousa P.A., Kulkarni S., Langille B.L., Zhu D.G., Davies T.C., Juneja S.C., Kidder G.M., Rossant J. (1995). Cardiac Malformation in Neonatal Mice Lacking Connexin43. Science.

[35] Gu H., Smith F.C., Taffet S.M., Delmar M. (2003). High incidence of cardiac malformations in connexin40-deficient mice. Circ. Res..

[36] Seki A., Ishikawa T., Daumy X., Mishima H., Barc J., Sasaki R., Nishii K., Saito K., Urano M., Ohno S. (2017). Progressive Atrial Conduction Defects Associated With Bone Malformation Caused by a Connexin-45 Mutation. J. Am. Coll. Cardiol..

[37] Munger S.J., Kanady J.D., Simon A.M. (2013). Absence of venous valves in mice lacking Connexin37. Dev. Biol..

[38] Kanady J.D., Munger S.J., Witte M.H., Simon A.M. (2015). Combining Foxc2 and Connexin37 deletions in mice leads to severe defects in lymphatic vascular growth and remodeling. Dev. Biol..

[39] Martins-Marques T., Catarino S., Goncalves A., Miranda-Silva D., Goncalves L., Antunes P., Coutinho G., Moreira A.L., Pires I.F., Girao H. (2020). EHD1 Modulates Cx43 Gap Junction Remodeling Associated With Cardiac Diseases. Circ. Res..

[40] Tribulova N., Knezl V., Okruhlicova L., Slezak J. (2008). Myocardial Gap Junctions: Targets for Novel Approaches in the Prevention of Life-Threatening Cardiac Arrhythmias. Physiol Res.

[41] Spach M.S., Starmer C.F. (1995). Altering the Topology of Gap-Junctions—A Major Therapeutic Target for Atrial-Fibrillation. Cardiovasc. Res..

[42] Kato T., Iwasaki Y.K., Nattel S. (2012). Connexins and Atrial Fibrillation Filling in the Gaps. Circulation.

[43] Wetzel U., Boldt A., Lauschke J., Weigl J., Schirdewahn P., Dorszewski A., Doll N., Hindricks G., Dhein S., Kottkamp H. (2005). Expression of connexins 40 and 43 in human left atrium in atrial fibrillation of different aetiologies. Heart.

[44] Polontchouk L., Haefliger J.A., Ebelt B., Schaefer T., Stuhlmann D., Mehlhorn U., Kuhn-Regnier F., De Vivie E.R., Dhein S. (2001). Effects of chronic atrial fibrillation on gap junction distribution in human and rat atria. J. Am. Coll. Cardiol..

[45] Kanagaratnam P., Cherian A., Stanbridge R.D.L., Glenville B., Severs N.J., Peters N.S. (2004). Relationship between connexins and atrial activation during human atrial fibrillation. J. Cardiovasc. Electr..

[46] Kostin S., Klein G., Szalay Z., Hein S., Bauer E.P., Schaper J. (2002). Structural correlate of atrial fibrillation in human patients. Cardiovasc. Res..

[47] Dhein S., Rothe S., Busch A., Rojas Gomez D.M., Boldt A., Reutemann A., Seidel T., Salameh A., Pfannmuller B., Rastan A. (2011). Effects of metoprolol therapy on cardiac gap junction remodelling and conduction in human chronic atrial fibrillation. Br. J. Pharmacol..

[48] Rucker-Martin C., Milliez P., Tan S., Decrouy X., Recouvreur M., Vranckx R., Delcayre C., Renaud J.F., Dunia I., Segretain D. (2006). Chronic hemodynamic overload of the atria is an important factor for gap junction remodeling in human and rat hearts. Cardiovasc. Res..

[49] Fakuade F.E., Tomsits P., Voigt N. (2021). Connexin hemichannels in atrial fibrillation: Orphaned and irrelevant?. Cardiovasc. Res..

[50] Dhein S., Salameh A. (2021). Remodeling of Cardiac Gap Junctional Cell-Cell Coupling. Cells.

[51] Jungk L., Franke H., Salameh A., Dhein S. (2019). Golgi Fragmentation in Human Patients with Chronic Atrial Fibrillation: A New Aspect of Remodeling. Thorac. Cardiov. Surg..

[52] Oyamada M., Takebe K., Oyamada Y. (2013). Regulation of connexin expression by transcription factors and epigenetic mechanisms. Biochim. Biophys. Acta.

[53] Ma J.F., Yang F., Mahida S.N., Zhao L., Chen X., Zhang M.L., Sun Z., Yao Y., Zhang Y.X., Zheng G.Y. (2016). TBX5 mutations contribute to early-onset atrial fibrillation in Chinese and Caucasians. Cardiovasc. Res..

[54] Mechakra A., Footz T., Walter M., Aranega A., Hernandez-Torres F., Morel E., Millat G., Yang Y.Q., Chahine M., Chevalier P. (2019). A Novel PITX2c Gain-of-Function Mutation, p.Met207Val, in Patients With Familial Atrial Fibrillation. Am. J. Cardiol..

[55] Yan J., Kong W., Zhang Q., Beyer E.C., Walcott G., Fast V.G., Ai X. (2013). c-Jun N-terminal kinase activation contributes to reduced connexin43 and development of atrial arrhythmias. Cardiovasc. Res..

[56] Wijffels M.C.E.F., Kirchhof C.J.H.J., Dorland R., Allessie M.A. (1995). Atrial-Fibrillation Begets Atrial-Fibrillation—A Study in Awake Chronically Instrumented Goats. Circulation.

[57] Rohr S. (2004). Role of gap junctions in the propagation of the cardiac action potential. Cardiovasc. Res..

[58] Kleber A.G., Rudy Y. (2004). Basic mechanisms of cardiac impulse propagation and associated arrhythmias. Physiol. Rev..

[59] Molica F., Merlijn J.P., Morel S., Kwak B.R. (2014). Mutations in cardiovascular connexin genes. Biol. Cell..

[60] Kanagaratnam P., Rothery S., Patel P., Severs N.J., Peters N.S. (2002). Relative expression of immunolocalized connexins 40 and 43 correlates with human atrial conduction properties. J. Am. Coll. Cardiol..

[61] Valiunas V., Gemel J., Brink P.R., Beyer E.C. (2001). Gap junction channels formed by coexpressed connexin40 and connexin43. Am. J. Physiol.-Heart Circ. Physiol..

[62] Gemel J., Levy A.E., Simon A.R., Bennett K.B., Ai X., Akhter S., Beyer E.C. (2014). Connexin40 abnormalities and atrial fibrillation in the human heart. J. Mol. Cell. Cardiol..

[63] Gutstein D.E., Morley G.E., Vaidya D., Liu F.Y., Chen F.L., Stuhlmann H., Fishman G.I. (2001). Heterogeneous expression of gap junction channels in the heart leads to conduction defects and ventricular dysfunction. Circulation.

[64] Firouzi M., Ramanna H., Kok B., Jongsma H.J., Koeleman B.P.C., Doevendans P.A., Groenewegen W.A., Hauer R.N.W. (2004). Association of human connexin40 gene polymorphisms with atrial vulnerability as a risk factor for idiopathic atrial fibrillation. Circ. Res..

[65] Juang J.M., Chern Y.R., Tsai C.T., Chiang F.T., Lin J.L., Hwang J.J., Hsu K.L., Tseng C.D., Tseng Y.Z., Lai L.P. (2007). The association of human connexin 40 genetic polymorphisms with atrial fibrillation. Int. J. Cardiol..

[66] Wirka R.C., Gore S., Van Wagoner D.R., Arking D.E., Lubitz S.A., Lunetta K.L., Benjamin E.J., Alonso A., Ellinor P.T., Barnard J. (2011). A Common Connexin-40 Gene Promoter Variant Affects Connexin-40 Expression in Human Atria and Is Associated With Atrial Fibrillation. Circ. Arrhythmia Electrophysiol..

[67] Christophersen I.E., Holmegard H.N., Jabbari J., Haunso S., Tveit A., Svendsen J.H., Olesen M.S. (2013). Rare Variants in GJA5 Are Associated With Early-Onset Lone Atrial Fibrillation. Can. J. Cardiol..

[68] Carballo S., Pfenniger A., Carballo D., Garin N., James R.W., Mach F., Shah D., Kwak B.R. (2018). Differential Association of Cx37 and Cx40 Genetic Variants in Atrial Fibrillation with and without Underlying Structural Heart Disease. Int. J. Mol. Sci..

[69] Gollob M.H., Jones D.L., Krahn A.D., Danis L., Gong X.Q., Shao Q., Liu X.Q., Veinot J.P., Tang A.S.L., Stewart A.F.R. (2006). Somatic mutations in the connexin 40 gene (GJA5) in atrial fibrillation. N. Engl. J. Med..

[70] Lubkemeier I., Andrie R., Lickfett L., Bosen F., Stockigt F., Dobrowolski R., Draffehn A.M., Fregeac J., Schultze J.L., Bukauskas F.F. (2013). The Connexin40A96S mutation from a patient with atrial fibrillation causes decreased atrial conduction velocities and sustained episodes of induced atrial fibrillation in mice. J. Mol. Cell. Cardiol..

[71] Yang Y.Q., Zhang X.L., Wang X.H., Tan H.W., Shi H.F., Jiang W.F., Fang W.Y., Liu X. (2010). Connexin40 nonsense mutation in familial atrial fibrillation. Int. J. Mol. Med..

[72] Sun Y.G., Tong X.L., Chen H.H., Huang T., Shao Q., Huang W.X., Laird D.W., Bai D.L. (2014). An atrial-fibrillation-linked connexin40 mutant is retained in the endoplasmic reticulum and impairs the function of atrial gap-junction channels. Dis. Model. Mech..

[73] Yang Y.Q., Liu X., Zhang X.L., Wang X.H., Tan H.W., Shi H.F., Jiang W.F., Fang W.Y. (2010). Novel connexin40 missense mutations in patients with familial atrial fibrillation. Europace.

[74] Sun Y.G., Yang Y.Q., Gong X.Q., Wang X.H., Li R.G., Tan H.W., Liu X., Fang W.Y., Bai D.L. (2013). Novel Germline GJA5/Connexin40 Mutations Associated with Lone Atrial Fibrillation Impair Gap Junctional Intercellular Communication. Hum. Mutat..

[75] Sun Y.G., Hills M.D., Ye W.G., Tong X.L., Bai D.L. (2014). Atrial Fibrillation-Linked Germline GJA5/Connexin40 Mutants Showed an Increased Hemichannel Function. PLoS ONE.

[76] Shi H.F., Yang J.F., Wang Q., Li R.G., Xu Y.J., Qu X.K., Fang W.Y., Liu X., Yang Y.Q. (2013). Prevalence and spectrum of GJA5 mutations associated with lone atrial fibrillation. Mol. Med. Rep..

[77] Noureldin M., Chen H.H., Bai D.L. (2018). Functional Characterization of Novel Atrial Fibrillation-Linked GJA5 (Cx40) Mutants. Int. J. Mol. Sci..

[78] Rudy Y. (2008). Molecular basis of cardiac action potential repolarization. Ann. N. Y. Acad. Sci..

[79] Dale N. (2008). Dynamic ATP signalling and neural development. J. Physiol..

[80] Paznekas W.A., Boyadjiev S.A., Shapiro R.E., Daniels O., Wollnik B., Keegan C.E., Innis J.W., Dinulos M.B., Christian C., Hannibal M.C. (2003). Connexin 43 (GJA1) mutations cause the pleiotropic phenotype of oculodentodigital dysplasia. Am. J. Hum. Genet..

[81] Kalcheva N., Qu J.X., Sandeep N., Garcia L., Zhang J., Wang Z.Y., Lampe P.D., Suadicani S.O., Spray D.C., Fishman G.I. (2007). Gap junction remodeling and cardiac arrhythmogenesis in a murine model of oculodentodigital dysplasia. Proc. Natl. Acad. Sci. USA.

[82] Dobrowolski R., Sasse P., Schrickel J.W., Watkins M., Kim J.S., Rackauskas M., Troatz C., Ghanem A., Tiemann K., Degen J. (2008). The conditional connexin43G138R mouse mutant represents a new model of hereditary oculodentodigital dysplasia in humans. Hum. Mol. Genet..

[83] Delmar M., Makita N. (2012). Cardiac connexins, mutations and arrhythmias. Curr. Opin. Cardiol..

[84] Thibodeau I.L., Xu J., Li Q.J., Liu G.L., Lam K., Veinot J.P., Birnie D.H., Jones D.L., Krahn A.D., Lemery R. (2010). Paradigm of Genetic Mosaicism and Lone Atrial Fibrillation Physiological Characterization of a Connexin 43-Deletion Mutant Identified From Atrial Tissue. Circulation.

[85] Kruger O., Plum A., Kim J.S., Winterhager E., Maxeiner S., Hallas G., Kirchhoff S., Traub O., Lamers W.H., Willecke K. (2000). Defective vascular development in connexin 45-deficient mice. Development.

[86] Kumai M., Nishii K., Nakamura K., Takeda N., Suzuki M., Shibata Y. (2000). Loss of connexin45 causes a cushion defect in early cardiogenesis. Development.

[87] Frank M., Wirth A., Andrie R.P., Kreuzberg M.M., Dobrowolski R., Seifert G., Offermanns S., Nickenig G., Willecke K., Schrickel J.W. (2012). Connexin45 Provides Optimal Atrioventricular Nodal Conduction in the Adult Mouse Heart. Circ. Res..

[88] Rackauskas M., Kreuzberg M.M., Pranevicius M., Willecke K., Verselis V.K., Bukauskas F.F. (2007). Gating properties of heterotypic gap junction channels formed of connexins 40, 43, 45. Biophys. J..

[89] Li R.G., Xu Y.J., Ye W.G., Li Y.J., Chen H.H., Qiu X.B., Yang Y.Q., Bai D.L. (2021). Connexin45 (GJC1) loss-of-function mutation contributes to familial atrial fibrillation and conduction disease. Heart Rhythm..

[90] Richard G., Lin J.P., Smith L., Whyte Y.M., Itin P., Wollina U., Epstein E., Hohl D., Giroux J.M., Charnas L. (1997). Linkage studies in erythrokeratodermias: Fine mapping, genetic heterogeneity, and analysis of candidate genes. J. Investig. Dermatol..

[91] Derouette J.P., Desplantez T., Wong C.W., Roth I., Kwak B.R., Weingart R. (2009). Functional differences between human Cx37 polymorphic hemichannels. J. Mol. Cell. Cardiol..

[92] Kumari S.S., Varadaraj K., Valiunas V., Ramanan S.V., Christensen E.A., Beyer E.C., Brink P.R. (2000). Functional expression and biophysical properties of polymorphic variants of the human gap junction protein connexin37. Biochem. Bioph. Res. Commun..

[93] Wong C.W., Christen T., Pfenniger A., James R.W., Kwak B.R. (2007). Do allelic variants of the connexin37 1019 gene polymorphism differentially predict for coronary artery disease and myocardial infarction?. Atherosclerosis.

[94] Katakami N., Sakamoto K., Kaneto H., Matsuhisa M., Shimizu I., Ishibashi F., Osonoi T., Kashiwagi A., Kawamori R., Hori M. (2009). Association between the connexin37 polymorphism and peripheral arterial disease in subjects with type 2 diabetes. Diabetes Care.

[95] Guo S.X., Yang Z.Y., Wang R.X., Yang Y., Cao H.M., Zhang T. (2013). Association between C1019T polymorphism of the connexin37 gene and coronary heart disease in patients with in-stent restenosis. Exp. Ther. Med..

[96] Listi F., Candore G., Balistreri C.R., Caruso M., Incalcaterra E., Hoffmann E., Lio D., Caruso C. (2007). Connexin37 1019 gene polymorphism in myocardial infarction patients and centenarians. Atherosclerosis.

[97] Leu H.B., Chung C.M., Chuang S.Y., Bai C.H., Chen J.R., Chen J.W., Pan W.H. (2011). Genetic variants of connexin37 are associated with carotid intima-medial thickness and future onset of ischemic stroke. Atherosclerosis.

[98] Wong C.W., Christen T., Roth I., Chadjichristos C.E., Derouette J.P., Foglia B.F., Chanson M., Goodenough D.A., Kwak B.R. (2006). Connexin37 protects against atherosclerosis by regulating monocyte adhesion. Nat. Med..

[99] Wen D., Du X., Nie S.P., Dong J.Z., Ma C.S. (2014). Association of Connexin37 C1019T with myocardial infarction and coronary artery disease: A meta-analysis. Exp. Gerontol..

[100] Zhao L., Li Y., Wu D., Ma T., Xia S.Y., Liu Z. (2014). Cx37 C1019T Polymorphism May Contribute to the Pathogenesis of Coronary Heart Disease. Genet. Test. Mol. Biomark..

[101] Kjolbye A.L., Knudsen C.B., Jepsen T., Larsen B.D., Petersen J.S. (2003). Pharmacological characterization of the new stable antiarrhythmic peptide analog Ac-D-Tyr-D-Pro-D-Hyp-Gly-D-Ala-Gly-NH2 (ZP123): In vivo and in vitro studies. J. Pharmacol. Exp. Ther..

[102] Shiroshita-Takeshita A., Sakabe M., Haugan K., Hennan J.K., Nattel S. (2007). Model-dependent effects of the gap junction conduction-enhancing antiarrhythmic peptide rotigaptide (ZP123) on experimental atrial fibrillation in dogs. Circulation.

[103] Ghazizadeh Z., Kiviniemi T., Olafsson S., Plotnick D., Beerens M.E., Zhang K., Gillon L., Steinbaugh M.J., Barrera V., Sui S.H. (2020). Metastable Atrial State Underlies the Primary Genetic Substrate for MYL4 Mutation-Associated Atrial Fibrillation. Circulation.

[104] Orr N., Arnaout R., Gula L.J., Spears D.A., Leong-Sit P., Li Q.J., Tarhuni W., Reischauer S., Chauhan V.S., Borkovich M. (2016). A mutation in the atrial-specific myosin light chain gene (MYL4) causes familial atrial fibrillation. Nat. Commun..

[105] O’Quinn M.P., Palatinus J.A., Harris B.S., Hewett K.W., Gourdie R.G. (2011). A Peptide Mimetic of the Connexin43 Carboxyl Terminus Reduces Gap Junction Remodeling and Induced Arrhythmia Following Ventricular Injury. Circ. Res..

[106] Salameh A., Apel D., Casanova J.G., von Salisch S., Mohr F.W., Daehnert I., Dhein S. (2012). On the different roles of AT(1) and AT(2) receptors in stretch-induced changes of connexin43 expression and localisation. Pflügers Arch. Eur. J. Physiol..

[107] Yamazaki T., Yazaki Y. (1999). Role of tissue angiotensin II in myocardial remodelling induced by mechanical stress. J. Hum. Hypertens..

[108] Zhang Y., Hou M.C., Li J.J., Qi Y., Zhang Y., She G., Ren Y.J., Wu W., Pang Z.D., Xie W. (2020). Cardiac beta-adrenergic receptor activation mediates distinct and cell type-dependent changes in the expression and distribution of connexin 43. J. Cell. Mol. Med..

[109] Lissoni A., Hulpiau P., Martins-Marques T., Wang N., Bultynck G., Schulz R., Witschas K., Girao H., De Smet M., Leybaert L. (2021). RyR2 regulates Cx43 hemichannel intracellular Ca2+-dependent activation in cardiomyocytes. Cardiovasc. Res..

[110] Igarashi T., Finet J.E., Takeuchi A., Fujino Y., Strom M., Greener I.D., Rosenbaum D.S., Donahue J.K. (2012). Connexin gene transfer preserves conduction velocity and prevents atrial fibrillation. Circulation.

